# The Adult Sensory Profile™ in Care Homes Targeting People Diagnosed with Dementia: A Qualitative Study from the Care Provider Perspective

**DOI:** 10.1155/2018/5091643

**Published:** 2018-08-05

**Authors:** Maiken Bay Ravn, Tinna Klingberg, Kirsten Schultz Petersen

**Affiliations:** ^1^Department of Health Science and Technology, Aalborg University, Aalborg, Denmark; ^2^Municipality of Aalborg, Denmark

## Abstract

**Introduction:**

The background of this study is the pilot testing of the Adolescent /Adult Sensory Profile (A/ASP) in dementia units at municipal nursing homes. Based on the results from therapists' A/ASP assessment, recommendations are made according to individual needs and forwarded to the health care providers. This study looks into the health care providers' perspective on the usability of these recommendations.

**Aim:**

The aim of this qualitative study is to explore the health care providers' perspective on the usability of recommendations derived from the A/ASP during a pilot testing of the profile in dementia units for people living with severe dementia.

**Methods:**

Participant observations and informal and formal interviews with health care providers at five municipal dementia units during the pilot testing of the A/ASP.

**Results:**

In the health care provider perspective, the A/ASP is a relevant and useful tool to use when behavioural challenges among residents living with dementia occur. However, in their opinion, it requires time, adjustment, and further education if recommendations are to be fully implemented in everyday practice at the dementia units.

## 1. Introduction

Dementia is a neurodegenerative syndrome resulting in functional, cognitive, and behavioural changes that may result in reduced ability to perform daily activities. The accuracy of sensory information registration is also reduced, affecting the perception and processing of sensory information [1]. Symptoms may vary from mood changes to depression, agitation, anxiety, wandering, and behavioural dysregulation [2–4]. The behavioural changes have an enormous impact on everyday lives of patients and relatives and can pose a challenge to health care providers [5]. Due to dementia being incurable, therapy and care aim to improve and maintain daily functioning [3, 5], and both pharmacological and nonpharmacological interventions are offered [6, 7].

Emotion-, behaviour-, and cognitive-oriented interventions and sensory stimulation are often used as nonpharmacological interventions [2, 6]. Sensory stimulation interventions aim to increase peoples quality of life (QoL) by stimulating the senses, increasing alertness, helping control behaviour, reducing agitation, and helping people feel safe [2, 3]. However, the ability to process and respond to sensory information is seldom taken into consideration when implementing sensory stimulating interventions [1]. This possesses a need to assess the individual sensory processing ability.

The Adolescent/Adult Sensory Profile (A/ASP) is an instrument developed by Brown and Dunn to assess the sensory ability [8]. The A/ASP is based on the Sensory Profile, a sensory-motor test investigating the sensory ability of children with dysfunctions such as autism [9]. A/ASP is a modified version of Sensory Profile and measures the sensory ability of individuals from 11 to 65 years of age [8]. The instrument is based on 60 items divided into six different categories: auditory, visual, smell/taste, touch, movement, and activity level. The evaluation identifies four sensory processing patterns: low registration, sensation seeking, sensory sensitivity, and sensation avoiding [10]. Based on the results from A/ASP, recommendations for rehabilitation can be made according to the individual needs. A study from Brown et al. (2000) supports the reliability and validity of the A/ASP as distinct constructs of sensory processing preferences. However, the study highlights that further exploration of target populations is needed [10].

The Municipality of Aalborg pilot tested in 2016 the A/ASP in dementia units in some of the municipal nursing homes. The aim of the pilot testing was to evaluate whether the recommendations derived from the A/ASP could be used to reduce and prevent behavioural challenges posed by people living with severe dementia and help develop a better work environment. Overall, the evaluation showed a significant reduction in behavioural changes for people living with dementia which had an impact on the work environment. However, in cases where the recommendations were poorly implemented, no or only little effects were reported. This posed the need for further investigation of health care providers' perspective on the usability of the A/ASP.

The aim of this qualitative study is to explore the health care providers' perspective on the usability of recommendations derived from the A/ASP during the pilot testing of the profile in dementia units for people living with severe dementia.

## 2. Method

The study was designed as a qualitative study with formal and informal individual interviews and participant observations at the dementia units involved in the pilot testing of the A/ASP at municipal nursing homes.

### 2.1. Settings

The pilot testing of the A/ASP took place from February to June 2016 at five dementia units. The included dementia units were specialised units for people living with severe dementia, housing eight till 15 residents that lived in a small apartment with dual living/bedroom, kitchenette, and bathroom.

During the pilot testing of A/ASP the sensory abilities of 30 residents were assessed by a physiotherapist or an occupational therapist. The assessment was based on information gathered from the primary health care providers, as the person with dementia had difficulties in answering questions about their sensory ability. Based on the assessment the physiotherapist or the occupational therapist made recommendations for each resident and presented these to the health care provider in charge of handing over the information to the rest of the staff.

### 2.2. Inclusion Criteria for Interviews

The inclusion criteria for both formal and informal interviews were health care providers that had been involved in the pilot testing of A/ASP and used the recommendations in their daily work with residents and with a minimum of 3 months' experience in using these recommendations.

### 2.3. Participants Involved in the Formal Interviews

Five health care providers were involved in the pilot testing of A/ASP, who had attended meetings with a therapist and were responsible for bringing forward the recommendations to their colleagues at the dementia unit. The participants were educated as health care helpers or health care assistants, with an education ranging in length from 14 months to 2 years and 9 months. Participants were 36 to 63 years of age.

### 2.4. Participants Involved in the Informal Interviews

Eight health care providers which had received the A/ASP recommendations from their colleague participated in the informal interviews during the observations made at the dementia units. All were educated as health care helpers or assistants.

### 2.5. Data Collection

The data collection took place from May till June 2016, while the pilot testing of the A/ASP took place. Data was collected over the duration of three to four visits which lasted three to four hours at each of the dementia units. The data collection involved participant observations and informal and formal individual interviews, lasting from 45 minutes till one hour. The interview guide used during the data collection was structured into three main themes:

1. What are your experiences in using recommendations from A/ASP?
2. Are there any challenges in using recommendations from A/ASP in your daily practices, and which?
3. What do you think are the future challenges in using recommendations from A/ASP?

Observations and informal interviews were used to gain a deeper understanding of the context in which the A/ASP was pilot tested. Open-ended questions were used: “How do you use the recommendations form A/ASP in your work?” and “How do the recommendations from the A/ASP affect the manner in which you work?” and focused questions, e.g., “I observed that …,” I noticed that…,” “Can you tell me what you did and how you have used the recommendations from the A/ASP?”

Participant observations were inspired by James Spradleys' approach to participant observation and planned in three phases: a descriptive, a focused, and a selective phase [11]. During the descriptive phase, an overall impression of the context for pilot testing the A/ASP was obtained, and field notes about the physical environment, the daily routines, and the interaction between health care providers and residents at the dementia units were taken. After a few visits at the dementia units, the more focused and selective phases entailed, observing the health care providers involved in using the recommendations from A/ASP in their daily work. In the selective phase, observations and formal interviews were conducted exclusively with the health care providers who were involved in the pilot testing and in charge of handling information to the rest of staff. Field notes from observations were read and used to plan the formal individual interviews of health care providers. These interviews aimed to gain a deeper understanding of the health care provider's individual experiences of using and bringing forward recommendations from A/ASP.

### 2.6. Analysis

The formal interviews of health care providers involved in meetings with therapists and in charge of bringing forward the recommendations to the rest of staff were recorded and transcribed verbatim. Field notes from observations and informal interviews with health care providers who received the recommendations from a colleague were used in the analysis. The analysis was inspired by Margrit Schreier's inductive and data-driven approach to content analysis [12]. A coding frame helped to distinguish between relevant and irrelevant parts by focusing only on data passages targeting the aim of the study. A matrix was used to illustrate and present the identified main categories with corresponding subcategories (Table 1).

### 2.7. Ethical Considerations

Oral informed consent was obtained from managers prior to the data collection at the dementia units. Written informed consent was obtained from the health care providers who participated in the formal interviews. The ethical guidelines as outlined in the Declaration of Helsinki were followed, ensuring anonymity and confidentiality of the participants and the residents at the dementia units. No personal information which could identify the involved persons was used. The last author who performed the data collection had previously worked as an occupational therapist at a dementia unit, which helped in interacting with residents during observations.

## 3. Results

To characterize the health care providers' perspective on the usability of recommendations derived from the A/ASP assessment, three main categories with corresponding subcategories emerged as a result of the analysis (Table 1).

In the health care provider's perspective, the recommendations received from the A/ASP assessment were experienced as follows:

1. An instrument for better understanding and managing residents with severe dementia.
2. Recommendations requiring time, adjustments, and knowledge.
3. A challenge to convey information to colleagues.

The overall experience of the usability of recommendations derived from the A/ASP in the dementia units was from the health care provider's perspective very positive. The health care providers found the recommendations to be useful and it helped manage residents with severe dementia and behavioural changes. Health care providers highlighted implications for practice and addressed day-to-day challenges and called for further knowledge about the A/ASP and how to apply the recommendations in practice.

### 3.1. An Instrument for Better Understanding and Managing Residents with Severe Dementia

The knowledge about the residents' sensory ability from the A/ASP gave health care providers a better understanding and gave them a tool for managing everyday life interactions with residents with severe dementia.

“We have become more aware of how we can observe and gain a better understanding of what is happening in his system” (INF5).

The health care providers saw potential to use the recommendations derived from the A/ASP on other residents with behavioural changes. The recommendations can in their perspective be used to interact with residents in an alternative manner and help understand why residents behave like they do. The recommendations derived from the A/ASP offered them new insights and gave them new ways to handle the behavioural changes of residents. Following the recommendations from A/ASP made it much easier for the health care providers to manage the residents during everyday activities such as meals and bathing.

“It is someone who has problems that we don't quite understand … by doing the A/ASP, we have moved much further towards better understanding what we can do; she is reacting to smells … she is coming further towards reality when there is something for her to smell … meatballs frying or flowers in a vase” (INF3).

Several of the health care providers found it surprising how residents responded to their new ways of interacting. From the health care providers' perspective, the use of physical force during the testing period had decreased as a result of using the recommendations derived from A/ASP. Additionally, the recommendations helped in creating a better relationship with residents and made everyday activities easier.

### 3.2. Recommendations Require Time, Adjustments, and Knowledge

The health care providers found using the recommendations from the A/ASP in their daily practices to be time-consuming. The recommendations derived from the A/ASP needed to be adjusted individually to each of the residents, which did not always work in practice. Making the residents perform daily activities, such as eating, bathing, and personal care, was time-consuming and, as a result, often posed a barrier for the health care providers. Frequently, it was necessary for two people to manage one resident, and colleagues were not always familiar with the A/ASP recommendations.

“It is slightly more demanding, but these are the conditions within the elderly sector – then, it is difficult to go in depth with it (A/ASP); there will be days during which it is simply not prioritized” (INF2).

When implementing changes, it was not necessarily appropriate to introduce several changes simultaneously. Each of the recommendations made by the A/ASP had to be adjusted to the actual mood and rhythm of the day. Both the health care providers and the residents became easily confused about the new practice, casting doubt on the usefulness and possible effects.

“It is highly dependent on how the day has been, how the night has been, there are oscillations, and it is the little things that are needed to not seem disheartened; that is particularly the picture for him, and for others, it is anxiety” (INF1).

Knowledge about the A/ASP is needed prior to using the recommendations derived from it. The health care providers did not always know the theory behind the recommendations unless they had been introduced to sensory stimulation on a previous occasion.

“Yeah, but I was not introduced to it very well. At first, you are told that there are these initiatives that you need to set into action. That is fine, but what is the background for it?” (INF2).

The health care providers highlighted that, apart from the pilot testing of the A/ASP, several other projects took their time. Additionally, they called for more information about the A/ASP and its use as they were in doubt about the usability of the recommendations. The health care providers who had attended meetings with therapists about the A/ASP felt more informed and better equipped to use the A/ASP recommendations compared to those who only received the recommendations via their colleagues.

### 3.3. A Challenge to Convey Information to Colleagues

The health care providers found it important that all staff at the dementia unit were informed about the recommendations derived from the A/ASP assessment. The recommendations were only to be distributed to staff if recommendations had to be used in daily practice. The health care providers involved in the testing of the A/ASP felt confident about using the recommendations in their daily practice, but they found it difficult to get enough time to convey information to others, as they were obliged to. The care plan was highlighted as a relevant platform for informing the health care providers about the following changes in how they should handle residents differently.

“Unfortunately, one feels that informing the team about what we do and why we do it so that everybody understands why we do the things we do provides extra work, and for me, who has been attending all the meetings … it is slightly easier to engage. However, I need to make the others feel that it is just as interesting and the extra effort makes sense and then, it requires that everyone finds it worth trying” (INF5).

Information about best practices and how to handle residents differently had to be conveyed to colleagues. Recommendations had to be informed to everybody at the dementia unit to be transferred into everyday life. The health care providers involved in the implementation of the recommendations found it difficult to distribute the information to all staff, including the health care providers working evenings and at night. However, when information was distributed to everyone at the unit, they experienced a change in the behaviour of the residents, and small changes in behavioural made, in their perspective, great differences in daily practice. The use of the A/ASP contributed to better communication with the residents.

“It has already spread (to health care providers), and this means that we also use it with other residents; even though we know that there are big differences from resident to resident, we can still try some of the same things, e.g., ball blankets and vests” (INF3).

The therapists conveyed recommendations to the health care provider, but, then, they had to inform the rest of staff about the possible changes in how to handle the residents. New and an excessive number of unfamiliar concepts were used to describe the residents' behaviour, so many of the health care providers called for further information about the A/ASP assessment.

“I sort of have to talk to my colleagues if it is also how they understand it … everyone has been fairly receptive to being a part of it; we are also a relatively small team” (INF5).

Sensory stimulation is not a common approach among health care providers at the dementia units involved in the pilot testing of the instrument. Despite the positive attitude to the use of new ways to handle residents in their daily practice, knowledge had to be transferred to all the staff at the dementia units in order to be able to understand how to use the recommendations in practice.

## 4. Discussion

The results from this qualitative study show that the health care providers find the recommendations derived from the A/ASP assessment to be useful and provide them with information on how to better understand and manage residents with severe dementia. Our results support that both individual factors, such as knowledge and skills, and contextual factors, such as time and workload, have an impact on how recommendations from A/ASP are implemented and used by health care providers at the dementia units. Despite the positive responses, challenges in implementing the recommendations occurred and recommendations derived from the A/ASP are not easily put into practice. Being able to both understand and manage residents with behavioural changes in new ways in daily practice at the dementia units requires time, knowledge, and skills in order to be able to make the necessary adjustments in the way that behavioural challenges of each individual resident are managed.

The three main categories in the health care providers' perspective on the use of the recommendations from the A/ASP assessment of residents with behavioural changes highlight issues to be addressed in the future implementation of the A/ASP at dementia units. Our study shows that health care providers which are receiving recommendations derived from the A/ASP need further knowledge about the A/ASP assessment and how to use the recommendations in practice.

In our study, the A/ASP was pilot tested on people with challenges in their behavioural performance as a result of suffering from severe dementia; its usability on this particular target group must be considered if it is to be implemented in the future. A systematic review by Strøm et al. (2016) found that the majority of studies regarding the sensory stimulation of people with dementia did not report the different levels of dementia [2]. Consequently, it may be difficult to locate relevant assessments to use in the rehabilitation of people with severe dementia. Further studies should focus on the development of interventions targeting severe stages of dementia and test their effectiveness. Another future challenge is the fact that the outcome measures used in the rehabilitation of people with dementia vary [2–4, 6, 13]. The various uses of outcome measures make transparency and comparability of studies difficult, thus impeding the implementation of new and more effective interventions to people with severe dementia. Cognitive rehabilitation interventions that target people with dementia involve a combination of approaches aimed at the restoration of function, the implementation of compensatory strategies, and environmental modification [14]. However, the preliminary results from the use of cognitive rehabilitation interventions show that it has the potential to cause changes in behaviour, enhance wellbeing, and maintain involvement in everyday life [14]; hence uncertainties of which interventions and assessments to use with people living with severe dementia continue to exist.

To our knowledge, no previous studies have explored health care providers' perspective of the usability of the A/ASP in people living with severe dementia. A review by Perkins (2012) found that education offered to health care providers helps reduce conflicts between health care providers and patients [3]. A randomized controlled trial by Clare et al. (2010) found that the effectiveness of interventions is increased when caregivers are actively involved in implementation [15]. Education and involvement of health care providers when implementing new interventions could help increase work satisfaction and decrease conflicts in dementia units for people with severe dementia.

Implementing new methods into practice involves, according to Dean Fixsen, six stages, of which the first stage, “investigation and adjustment,” is relevant to pay special attention to when implementing the A/ASP in dementia units. Core implementation components involve coaching and performance measurement [16]. In our study health care providers involved in testing the recommendations derived from A/ASP expressed the need to receive training in the A/ASP and further information about the theory and rationale behind sensory stimulation. Particularly the health care providers which were handed over the recommendations via their colleague called for further knowledge. Coaching during the implementation of recommendations from A/ASP in practice could be a way to meet health care provider needs. According to Dean Fixsen, performance measurement can help refine and qualify the implementation of new methods in practice [16]. The municipality in which this qualitative study took place concluded, after pilot testing the A/ASP that if it is to be implemented in dementia units, the following components had to be considered: organization, training of staff, and financing as both the assessment and the knowledge transfer are time-consuming. A panel of experts is currently working on how to organize the intervention and formulate a plan for training and calculating the possible costs.

This study of health care providers' perspective on the use of recommendations from A/ASP has some limitations. The small sample size has to be taken into account as well as the fact that pilot testing of an assessment in English also involves translation and fidelity testing of the instrument. Despite the study limitations, the strength of the study is the insight it gives about its implications for practice and issues raised by health care providers which have to be considered in future implementation of the assessment in dementia care. In addition, the transferability of findings has to be taken into account, due to the small sample size and the particular context in which the pilot testing took place. Future studies on the feasibility of the instrument are needed in order to be able to implement the A/ASP on people living with severe dementia.

## 5. Conclusion

This qualitative study, which followed the pilot testing of the A/ASP in dementia units for people living with severe dementia, shows that in the health care providers' perspective the recommendations received from A/ASP assessment are a useful tool and help to better understand and manage changes in behaviour. However, when implementing a new instrument such as the A/ASP in daily practice in the dementia units, several considerations should in the health care providers' perspective be taken into account. From the their perspective, it requires both time and further knowledge about the A/ASP assessment and guidelines on how to convey information to colleagues involved in the daily care are of key concern. Furthermore, providing health care providers with the knowledge and skills to manage behavioural changes of residents living in dementia units could help increase the quality of life for residents and prevent conflicts due to the behavioural changes coursed by dementia.

## Figures and Tables

**Table 1:** Identified categories, corresponding subcategories, and quotes.

**Categories**	**Subcategories**	**Quotes illustrating categories**
A instrument for better understanding and managing residents with severe dementia	Understanding	“We have become more aware of how we can observe and gain a better understanding of what is happening in his system” (INF5).
	Managing	“It is someone who has problems that we don't quite understand … by doing that Sensory Profile, we have moved much further towards better understanding what we can do; she is reacting to smells … she is coming further towards reality when there is something for her to smell … meatballs frying or flowers in a vase” (INF3).

Recommendations requires time, adjustment and knowledge	Time	“It is slightly more demanding, but these are the conditions within the elderly sector – then, it is difficult to go in depth with it (ASP); there will be days during which it is simply not prioritized” (INF2).
	Adjustment	“It is highly dependent on how the day has been, how the night has been, there are oscillations, and it is the little things that are needed to not seem disheartened; that is particularly the picture for him, and for others, it is anxiety” (INF1).
	Knowledge	“Yeah, but I was not introduced to it very well. At first, you are told that there are these initiatives that you need to set into action. That is fine, but what is the background for it?” (INF2).

A challenge to convey information to colleagues	Receiving information	“Unfortunately, one feels that informing the team about what we do and why we do it so that everybody understands why we do the things we do provides extra work, and for me, who has been attending all the meetings … it is slightly easier to engage. However, I need to make the others feel that it is just as interesting and the extra effort makes sense and then, it requires that everyone finds it worth trying” (INF5).
	Transfer of information	“It has already spread (to health care providers), and this means that we also use it with other residents; although we know that there are big differences from resident to resident, we can still try some of the same things, e.g., ball blankets and vests” (INF3). “I sort of have to talk to my colleagues if it is also how they understand it … everyone has been fairly receptive to being a part of it; we are also a relatively small team” (INF5).

## Data Availability

The data used to support the findings of this study are available from the corresponding author upon request.
